# Glycogen availability and skeletal muscle adaptations with endurance and resistance exercise

**DOI:** 10.1186/s12986-015-0055-9

**Published:** 2015-12-21

**Authors:** Pim Knuiman, Maria T. E. Hopman, Marco Mensink

**Affiliations:** Division of Human Nutrition, Wageningen University, Bomenweg 4, 6703 HD Wageningen, The Netherlands; Radboud University, Radboud Institute for Health Sciences, Department of Physiology, Geert Grooteplein-West 32, 6525 GA Nijmegen, The Netherlands

**Keywords:** Glycogen availability, Skeletal muscle, Adaptation, Endurance exercise, Resistance exercise

## Abstract

It is well established that glycogen depletion affects endurance exercise performance negatively. Moreover, numerous studies have demonstrated that post-exercise carbohydrate ingestion improves exercise recovery by increasing glycogen resynthesis. However, recent research into the effects of glycogen availability sheds new light on the role of the widely accepted energy source for adenosine triphosphate (ATP) resynthesis during endurance exercise. Indeed, several studies showed that endurance training with low glycogen availability leads to similar and sometimes even better adaptations and performance compared to performing endurance training sessions with replenished glycogen stores. In the case of resistance exercise, a few studies have been performed on the role of glycogen availability on the early post-exercise anabolic response. However, the effects of low glycogen availability on phenotypic adaptations and performance following prolonged resistance exercise remains unclear to date. This review summarizes the current knowledge about the effects of glycogen availability on skeletal muscle adaptations for both endurance and resistance exercise. Furthermore, it describes the role of glycogen availability when both exercise modes are performed concurrently.

## Background

Roughly, exercise can be divided in endurance- and resistance exercise. Endurance exercise can be further subdivided in traditional -endurance exercise and high intensity interval training (HIIT). Traditional endurance exercise is characterized by continues submaximal muscular contractions aimed at improving aerobic power production. Whereas high intensity interval training primarily consists of brief, intermittent bursts of vigorous movements, alternated by periods of rest or low-intensity movements with the purpose to improve both aerobic and anaerobic power production [1]. Resistance exercise, on the other hand involves short bursts of nearly maximal muscular contractions and primarily focuses on the development of muscle hypertrophy and/or muscular strength. The skeletal muscle adaptations are determined by the type, intensity and duration of the performed exercise. In short, endurance exercise training mainly results in mitochondrial biogenesis, increases capillary density and enzymes leading to enhanced skeletal muscle O_2_ utilization capacity [2–4]. In contrast, resistance exercise promotes skeletal muscle hypertrophy and strength through increases in myofibrillar volume predominantly in type II fibers [5, 6].

It is now widely accepted that nutrition plays an important role in mediating skeletal muscle adaptations [7]. Carbohydrates and fat are recognized as the main substrates for powering prolonged muscle contractions during endurance exercise [8]. Although carbohydrates are widely accepted as fuel for skeletal muscle both during [8] and following endurance exercise [8], recent investigations introduced a novel approach of exercising with reduced glycogen levels aimed to optimize skeletal muscle adaptations [9, 10]. Indeed, several studies have reported that endurance exercise with low glycogen availability may be a strategy to augment the response in exercise-induced signaling associated with improved oxidative capacity [11–17], and potentially enhance exercise performance [17, 18]. In contrast, the effects of low glycogen availability on muscular adaptations following resistance exercise remain somewhat unclear. A recent study revealed that performing resistance exercise with low glycogen could improve acute signaling processes that promote mitochondrial biogenesis to a larger extent compared to exercise with normal glycogen levels [19], whereas another study demonstrated that muscle protein synthesis following a single bout of resistance exercise appeared to be unaffected by the level of glycogen [20].

A literature review concerning the role of glycogen availability for both endurance- and resistance exercise on skeletal muscle adaptations is at this time absent. Therefore, the purpose of this review is to identify the effects of glycogen availability on skeletal muscle training adaptations and performance with both endurance- and resistance exercise. Firstly, the role of glycogen in local skeletal muscle fatigue and energy metabolism will be described. Thereafter, the effects of glycogen availability on performance and markers of skeletal muscle adaptations are discussed. Finally, this review addresses the role of glycogen availability when both exercise modes are performed concurrently.

### Glycogen content, location and skeletal muscle fatigue

In humans, most glycogen is made and stored in cells of the liver (~100 g) and muscles (~350 – 700 g; depending on training status, diet, muscle fibre type composition, sex and bodyweight) and can be reduced by fasting, low intake of dietary carbohydrates and/or by exercise. It seems that the critical level of muscle glycogen is approximately around 250-300 mmol∙kg^-1^dry weight (d.w.), levels below this amount have been associated with impaired sarcoplasmic reticulum function by diminishing vesicle Ca^2+^ release rate and reductions in peak power output [21]. Glycogen is differently distributed within the muscle fibers (subsarcolemmal ~5-15 %, intermyofibrillar ~75 % and intramyofibrillar ~5-15 %) [22]. Moreover, it appears that subsarcolemmal, intermyofibrillar and intramyofibrillar glycogen powers different mechanisms in muscle contractions. It is thought that intermyofibrillar glycogen powers the release of sarcoplasmic stored Ca^2+^ and in this way activates the tropomyosin active sites. Intramyofibrillar glycogen is preferably depleted during high-intensity exercise and seems to power cross-bridge cycling [23]. Moreover, depletion of this form highly correlates well with skeletal muscle fatigue [24]. Reduction of intramyofibrillar glycogen might decrease Na, K-ATPase activity leading to decreased ATP cleavage, and subsequently a lower energy production to power cross-bridge cycling [22].

Moreover, Duhamel et al. [25] found that commencing an endurance type bout of exercise till fatigue with low glycogen availability resulted in earlier deteriorations in SR Ca^2+^ release. Specifically, their data indicated that a cycling session of 70 % VO_2peak_ carried out at low glycogen levels causes faster reductions in SR Ca^2+^ uptake and Ca^2+^ release during exercise compared to high glycogen levels. Furthermore, it was found that reductions in SR Ca^2+^ -ATPase activity followed a similar time course as that of Ca^2+^ uptake suggesting a mediating role for SR Ca^2+^ -ATPase activity. In another study by Ortenblad et al. [21] it was shown that ingestion of carbohydrates during 4 h recovery following exercise markedly increases glycogen content and normalizes SR Ca^2+^ compared to the group who were omitted from carbohydrates during the recovery period. Based on SR vesicle experiments Ortenblad et al. [22] proposed that there is mechanistic role of glycogen on SR Ca^2+^ release. Moreover, Ortenblad et al. stated that the reduction in SR Ca^2+^ release by itself induces a diminution in tetanic intracellular free [Ca^2+^]_i_, which is in accordance with isolated fibres studies showing a faster decrease of tetanic [Ca^2+^]_i_ when glycogen content is reduced within these fibres. Taken together, the aforementioned findings at both the whole-body and organelle level suggest that the location of the glycogen, especially the intramyofibrillar pool, is important to sustain repeated muscle contractions.

### Glycogen and energetic demands with exercise

Glycogen is an essential substrate during high intensity exercise by providing a mechanism by which adenosine tri phosphate (ATP) can be resynthesized from adenosine diphosphate (ADP) and phosphate. The relative use of energy sources during exercise is mainly determined by the intensity and the duration of the exercise bout, as well as the athlete’s training status [8]. Fat as source of energy is relatively most dominant during moderate intensity (30-65 % of VO_2peak_), whereas the relative contribution of carbohydrate oxidation to total energy expenditure becomes greater when exercise intensity increases, with muscle glycogen becoming the most important substrate source [26]. Although the amount of liver and skeletal muscle glycogen is relatively small compared to endogenously stored fat, glycogen is recognized as the major source for fuel during prolonged moderate- to high intensity endurance exercise [27]. Therefore, glycogen availability is essential to power ATP resynthesis during high intensity exercise which relies heavily on glycogenolysis. Furthermore, it has been well documented that the capability of skeletal muscle to exercise is impaired when the glycogen store is reduced to a certain level, even when there is sufficient amount of other fuels available [28]. Together, prolonged endurance exercise leads to muscle glycogen depletion, which is in turn linked to fatigue and makes it difficult to meet the energetic requirements of training and competition [22, 29].

### Low glycogen and performance with exercise

#### Endurance training performance

Low-glycogen availability causes a shift in substrate metabolism during and after exercise [30, 31]. In addition, low-glycogen availability induces an increase in systemic release of amino acids and simultaneously increases fat oxidation, and as a consequence exercise intensity drops [30]. However, the low-glycogen approach seems to promote expression of genes that stimulate fat catabolism and mitochondrial biogenesis and as such improves oxidative capacity [10]. To date, few studies have found an improved training-induced performance effect of conducting the exercise bouts with low glycogen levels compared with replenished glycogen levels [17, 18]. Hansen et al. [17] were the first to show that training with reduced glycogen availability results in improved oxidative capacity. In their study seven untrained males completed a 10-week training program. The untrained subjects performed leg-knee extensor exercise for 5 d∙wk^-1^. Although the total amount of work was the same for each leg, one leg was trained in a glycogen depleted manner, while the contralateral leg was trained with full glycogen stores. The finding of their study was a significant gain in endurance (time till exhaustion) in the low-glycogen compared to normal glycogen levels. In addition, they found that low-glycogen improved oxidative capacity (citrate synthase activity) to a larger extent than commencing all exercise sessions with high-glycogen. The findings of Hansen et al. [17] were pioneering and in contrast with the studies reporting that glycogen content is a limiting factor when it comes to exercise adaptation and performance. Subsequently, other research groups tested the same hypothesis by using an alternative model with trained subjects [12, 16]. Yeo et al. [16] demonstrated that subjects who undertook the exercise sessions in the low-glycogen state (~50 % depletion) showed significantly lower performance during the sessions compared to the subjects that undertook the sessions with high-glycogen [16]. Interestingly, following the 3-wk intervention period, several markers of training adaption were increased. However, 60-min time-trial performance was similar in both the low-glycogen and high-glycogen group. Although speculative, the similar effect in performance suggests that the low-glycogen group showed a greater training adaptation, relative to their level of training intensity. Hulston et al. [12] reported that lipid oxidation during the steady-state exercise at 70 % VO_2max,_ increased more in the low-glycogen group relative to the high-glycogen group, as a result of increased intramuscular triglycerides utilization. Moreover, this was accompanied by increases in oxidation of fatty acids, sparing of muscle glycogen, and greater increases in succinate dehydrogenase and 3-hydroxyacyl-CoA dehydrogenase enzyme activity [12]. However, with regard to performance, the training with low muscle glycogen availability was not more effective than training with high muscle glycogen levels [12]. Together, low-glycogen availability affects substrate use during exercise by increasing fatty acid oxidation compared to training with normal glycogen levels; this effect is independent of the subject training status.

Recently, Cochran et al. [18] demonstrated that a two week HIIT protocol performed with low-glycogen, improved cycling time trial performance compared with high glycogen stores. Specifically, training sessions consisted of 5 times 4-min intervals at 60 % W_peak_ (~95-100 % of heart rate reserve) interspersed with 2 min of rest. Both groups trained on a total of 6 d over a 2-wk period, with a minimum of one day of rest between training days. Furthermore, subjects completed two identical HIIT sessions on each training day, separated by 3 h of recovery. After two weeks of HIIT, mean power output during a 250-kJ time trial increased to a greater extent in the low-glycogen group compared to the high-glycogen group [18]. A novel aspect of their study was that the subjects performed whole-body exercise for a relatively short period of time (2 weeks), while the study of Hansen et al. [17] lasted 10 weeks and used an exercise protocol where upper leg muscles performed isolated knee extensions.

#### Discrepancies between and limitations of the low-glycogen endurance exercise studies

A possible explanation for the different outcomes on performance between low-glycogen studies could be differences in the training status of the subjects. Hansen et al. [17] and Cochran et al. [18] used untrained subjects, whereas others tested well-trained subjects [12, 16]. Indeed, it has previously been shown that the effectiveness of nutritional interventions is influenced by the subject training status [32], possibly because trained subjects depend less on carbohydrate utilization because they have greater metabolic flexibility. Another methodological issue is the selected test used to determine performance. In some studies, self-selected intensities were used, which could be influenced by carbohydrate manipulation. Cochran et al. [18] therefore prescribed and controlled power output during training to ensure that glycogen manipulation did not affect training intensity.

To summarize, although some studies reported that repetitive low-glycogen training leads to improved performance compared with high glycogen [17, 18], extrapolating these findings to sports-specific performance should be done with prudence. First, the study of Hansen et al. [17] used an isolated knee-extensor model (5 d∙wk^-1^) as training protocol and performance measurement. However, this model does not accurately reflects an athlete’s performance in a real life sports event. Second, as suggested by Yeo et al. [16], athletes generally use multiple intensities, as well as progressive overload [33, 34], rather than a fixed submaximal exercise intensity as was used during the training sessions in the study of Hansen et al. [17]. Lastly, chronic exercise sessions commencing in the low-glycogen state may enhance the risk for overtraining syndrome [35] which in turn may result in reduced training capacity [36].

#### Resistance exercise performance

Resistance exercise is typically characterized by short bursts of nearly maximal muscular contractions. When performing resistance exercise, glycogen is crucial to resynthesize the phosphate pool, which provides energy during high intensity muscle contractions [37]. According to MacDougall et al. [37], the majority of ATP is derived from glycolysis [38]. In line with this, a typical resistance exercise session has been shown to reduces glycogen levels by approximately ~24-40 % [37, 39–41]. This reduction in glycogen content during exercise is determined by the duration, intensity and volume of the performed exercise bout. The largest reductions in glycogen are seen with high repetitions with moderate load training [40], an effect that mainly occurs in type II fibers [39]. It has been demonstrated that a reduction of muscle glycogen affects both isokinetic torque [29] and isoinertial resistance exercise capacity negatively [42]. However, this effect is not always evident [43] and is likely to be affected by the protocol used to induce glycogen depletion [44]. Based on the assumption that pre-exercise glycogen content can influence exercise performance, it seems that the pre-exercise carbohydrate ingestion requires particular attention [44].

Although it is widely accepted that carbohydrate ingestion before endurance exercise enhances work capacity [45, 46], carbohydrate ingestion before resistance exercise has not been studied to the same extent. The importance of carbohydrates for the resistance exercise-type athlete can be substantiated by the idea that glycogen plays a relatively important role in energy metabolism during resistance exercise. For example, it has been shown that pre-resistance exercise carbohydrate ingestion increases the amount of total work [47–49]. In contrast, other reports show no benefit of carbohydrate ingestion on total work capacity [50, 51]. To precisely determine the role of glycogen availability for the resistance exercise athlete more training studies that feature a defined area of outcome measures specifically for performance and adaptation are needed.

### Mitochondrial biogenesis on low-glycogen regimes and molecular pathways involved

#### Endurance exercise

##### PGC-1α

**Fig. 1:**
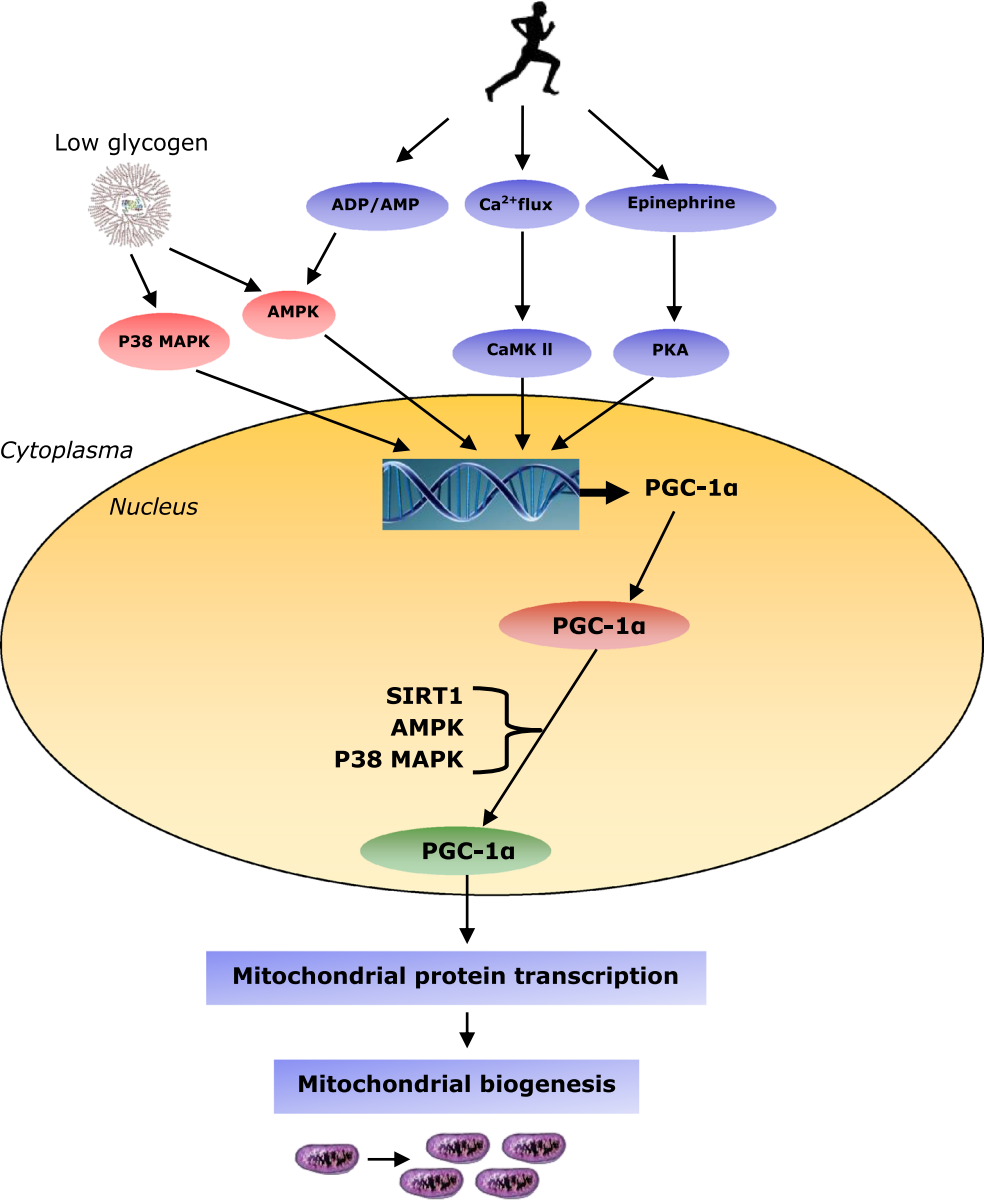
Schematic figure representing the regulation of mitochondrial biogenesis by endurance exercise (). Endurance exercise acutely increases Ca2+, ADP, AMP and epinephrine. In addition exercise reduces skeletal muscle glycogen () in the contracting muscles which in turn activates the sensing proteins AMPK and p38 MAPK. Especially elevated AMP and ADP trigger an increased phosphorylation of AMPK at Thr172 and the increased Ca2+ concentration via calmodulin causes CaMK II autophosphorylation. Both AMPK and p38 MAPK activate and translocate the transcriptional co-activator PGC-1α to the mitochondria and nucleus. The kinases AMPK, p38 MAPK and SIRT 1 then might phosphorylate PGC-1 α and reduce the acetylation of PGC-1 α, which increases its activity. Thus, endurance exercise leads to more PGC-1 α which over time results in mitochondrial biogenesis

Activity of the exercise-induced peroxisome proliferator-activated γ-receptor co-activator 1α (PGC-1α) has been proposed to play a key role in the adaptive response with endurance exercise (Fig. 1). Enhanced activity of PGC-1α and increased mitochondrial volume improves oxidative capacity through increased fatty acid *β*-oxidation and mitigating glycogenolysis [52]. As a result, muscle glycogen can be spared which might delay the onset of muscle fatigue and enhances oxidative exercise performance. PGC-1α is responsible for the activation of mitochondrial transcription factors e.g. the nuclear respiratory factors (NRF-1 and -2) and the mitochondrial transcription factor A (Tfam) [53].

##### PGC-1α and AMPK

Activation of PGC-1α is amongst others regulated by the major up-stream proteins 5' adenosine monophosphate-activated protein kinase (AMPK) [54]. Prolonged endurance type exercise requires a large amount of ATP resulting in accumulation of ADP and AMP in the recruited muscle fibers [55]. This activates AMPK with the purpose to restore cellular energy homeostasis [56, 57]. A relative drop of ATP by less than 1 % translates into a doubling or more of the ADP and AMP concentrations [58]. The rise of ADP and AMP during prolonged endurance type exercise results in the phosphorylation of AMPK at Thr172, the active site on the AMPK α subunit [58–60]. Canto and colleagues (2009) showed that AMPK action on PGC-1α transcriptional activity is partly regulated by SIRT1, a sirtuin family protein which deacetylates several proteins that contribute to cellular regulation [57]. Furthermore, it was shown that the acute actions of AMPK on lipid oxidation alter the balance between cellular NAD1 and NADH, which acts as a messenger to activate SIRT1 [57].

##### PGC-1 α, AMPK and p38 MAPK in response to glycogen depletion

During prolonged endurance type exercise skeletal muscle glycogen reduces, this is sensed by the AMPK β subunit resulting in an activation of AMPK (Fig. 1). The AMPK is then also activated through phosphorylation of Thr172 and this response is likely dependent on the rise of AMP and ADP during exercise. Chan et al (2004) suggested that low muscle glycogen availability associates with the phosphorylation of the nuclear P38 mitogen-activated protein kinases (p38 MAPK), rather than translocation of p38 MAPK to the nucleus per se [61]. Accordingly, p38 MAPK particularly phosphorylate the expression of PGC-1α [53, 62], whereas AMPK could both phosphorylate and enhance expression of PGC-1α [53, 62]. Restricted CHO availability during or after exercise has also been shown to augment phosphorylation of (i.e. activate) p38 MAPK [63] and AMPK [15]. In another study by Mathai and colleagues (2008) it was shown that changes in muscle glycogen correlates with the changes in PGC-1α protein abundance during exercise and recovery [64]. The majority of the studies show that the PGC-1α mRNA content increased during and directly after exercise and returned to resting levels by 24 h after exercise. However, the studies that measured both PGC-1α mRNA and PGC-1α protein after chronic or acute exercise failed to find increases in both [64]. Therefore, changes of PGC-1α mRNA content are not necessarily compatible with changes in PGC-1α protein abundance following exercise [64].

Although these studies suggest that the signalling response to exercise is affected by CHO supply, it remains unclear whether exercise in a glycogen-depleted state can enhance the adaptive signalling response that is required for mitochondrial biogenesis. Thus, AMPK and MAPK 38 play a key role in the transcriptional regulation of mitochondrial biogenesis trough PGC-1α in response to stress. However, the precise role of potential regulators which are responsive to glycogen availability, in the processes of mitochondrial biogenesis, needs to be further elucidated.

##### P53

Another described protein that regulates mitochondrial biogenesis is p53, which appears to be sensitive to changes in glycogen availability [65]. Previous research has shown that p53 is phosphorylated by AMPK and p38 AMPK [66, 67]. Furthermore, p53 is implicated in the stimulation of gene expression of mitochondrial function [66, 67]. It has been demonstrated that commencing endurance exercise in a glycogen depleted state upregulates p53 to a larger extent than during exercise in a replenished glycogen state [68]. However, the influence on PGC-1α mRNA expression is difficult to interpret because the subjects involved were not only on an exercise regime with low glycogen availability, but also on a calorie restricted diet. Accordingly, it remains unknown which potent regulator was responsible for the increase in mitochondrial biogenesis in this study. The precise role of both potential regulators in the processes of mitochondrial biogenesis needs to be further elucidated.

#### Resistance exercise

Although resistance exercise is mainly recognized as mechanical stimulus for increases in strength and hypertrophy, the aerobic effects following resistance exercise have also been studied. Early investigations have shown that skeletal mitochondrial volume [69] and oxidative capacity [70] are unaltered following prolonged resistance exercise. However, it has been recently reported that resistance exercise increases the activity of oxidative enzymes in tissue homogenates [19, 71] and respiration in skinned muscle fibers [72]. Moreover, resistance training augmented oxidative phosphorylation in sedentary older adults [73] and respiratory capacity and intrinsic function of skeletal muscle mitochondria in young healthy men [74]. In a recent investigation by Irving and co-workers [75] young and older adults performed 8 weeks of endurance training, resistance training or concurrent/combined training. Interestingly, following all exercise modalities, concurrent training induced the most robust improvements in mitochondrial related outcomes and mRNA expression [75].

Notably, the improvements in mitochondria were independent of age. Therefore, exploring molecular processes regulating the metabolic and oxidative responses with resistance training may lead to a better understanding and eventually to optimized adaptations. Studies examining the effect of low glycogen availability on mitochondrial regulators largely centered on endurance training. However, Camera et al. [19] recently showed that both phosphorylation of p53 and mRNA abundance of PGC-1α increased during the early (4 h) post-exercise recovery period after resistance exercise undertaken with low glycogen availability. It appears that the level of glycogen acts as a modulator of processes regulating mitochondrial biogenesis, independent of the nature of exercise stimuli. The supposed mechanism by which p53 is translocated from the nucleus to the mitochondria and subsequently enhances mitochondrial biogenesis is through its interaction with mitochondrial transcription factor A (Tfam) and also by preventing p53 suppression of PGC-1α activation in the nucleus [67]. According to the findings of Camera et al. [19] and others [20, 76, 77] resistance exercise seems capable of increasing PGC-1 mRNA expressions and p53 phosphorylation that has the potential to stimulate mitochondrial adaptation. Moreover, the acute metabolic response to resistance exercise can be modulated in a glycogen-dependent manner. However, whether these acute alterations in regulators of mitochondrial biogenesis are sufficient to promote mitochondrial volume and function remains to be elucidated in future long-term training studies.

### Low glycogen availability and protein metabolism

#### Endurance exercise

Skeletal muscle mass is maintained by the balance between muscle protein synthesis (MPS) and muscle protein breakdown (MPB) rates such that overall net muscle protein balance (NPB) remains essentially unchanged over the course of the day. A prolonged imbalance between MPS and MPB results in a positive (hypertrophy: MPS > MPB) or negative NPB (atrophy: MPS < MPB). The two main potent stimuli for MPS are food ingestion and exercise [78]. Nutrition, proteins in particular, induces a transient stimulation of MPS and is therefore in itself, i.e. in the absence of exercise, not sufficient to induce a positive NPB. Likewise, resistance exercise improves NPB, however, the ingestion of protein during the post-exercise recovery period is required to induce a positive NPB [79]. Thus, both exercise and food ingestion must be deployed in combination in order to create a positive NPB [78].

To date, only a few studies examined the role of glycogen availability on protein metabolism following endurance exercise [30, 80, 81]. It seems that glycogen availability mediates MPB. An early study from Lemon and Mullin showed that when exercise was performed with reduced glycogen availability nitrogen losses more than doubled, suggesting an increase in MPB and amino acid oxidation [80]. Subsequently, two other studies [30, 81] used the arterial-venous (a-v) difference method to explore whether exercise in the low glycogen state affects amino acid flux and then estimated NPB. In both studies subjects performed an exercise session in the low-glycogen state, the researchers found a net release of amino acids during exercise indicating an increase in MPB. However, these studies may be methodologically flawed because the a-v balance method only allows for the determination of net amino acid balance. Conclusions about changes in MPS and MPB are therefore of a speculative nature [82]. A more recent study by Howarth et al. [82] used the stable isotope tracer methodology and therefore enabled to determine the changes in MPS and MPB. They found that skeletal muscle NPB was lower when exercise was commenced with low glycogen availability compared to the high glycogen group, indicating an increase in MPB and decrease in MPS during exercise. It appears that endurance exercise with reduced muscle glycogen availability negatively influences muscle protein turnover and impairs skeletal muscle repair and recovery from endurance exercise. As described previously, low glycogen could be used as a strategy to augment mitochondrial adaptations to exercise, however, protein ingestion is required to offset MPB and increase MPS. Indeed, recent evidence reported that protein ingestion during or following endurance exercise increases MPS leading to a positive NPB [83, 84].

#### Resistance exercise

Resistance exercise type muscular contraction and/or protein ingestion affect the complex of regulatory processes that determines the changes in MPB and MPS. The Akt-mTOR-S6K pathway that controls the process of MPS has been studied extensively [85, 86]. However, the effects of glycogen availability with resistance exercise and its effects on these regulatory processes remains to be further scrutinized. It has been observed that low-glycogen availability (~160 mmol∙kg^-1^d.w.) elevates resting and exercise-induced AMPK activity compared to high-glycogen availability (~910 mmol∙kg^-1^d.w.) [87]. Furthermore, work by Churchly et al. [88] demonstrated that low-glycogen availability (~193 mmol∙kg^-1^d.w.) did not enhance the activity of genes involved in muscle hypertrophy. Creer et al. [89], on the other hand, investigated the role of glycogen availability on two key pathways involved in cellular growth. Subjects performed three sets of 10 repetitions of bilateral knee extension exercise at 70 % of 1-RM separated by a 2-min recovery period. Muscle biopsies were taken to determine the activation of extracellular signal-regulated kinase (ERK1/2) and Akt signaling pathways. They found an increase in both ERK1/2 and p90 ribosomal S6 kinase phosphorylation, but this effect was independent of the level of glycogen. Further, Akt phosphorylation was attenuated in the low glycogen (~175 mmol∙kg^-1^d.w.), whereas it was increased in the high glycogen (~600 mmol∙kg^-1^d.w.) group. mTOR phosphorylation was similar to that of Akt, however, the change was not significant. In a comparable study from Camera et al. [20] young healthy men performed 8 sets of 5 unilateral leg press repetitions at 80 % 1RM. Muscle biopsies were taken at rest and 1 and 4 h after the single exercise bout. Although mTOR phosphorylation increased to a higher extent in the normal glycogen group, there were no detectable differences found in MPS suggesting that the small differences in signaling are negligible since MPS was unaffected. The result that MPS is not influenced by the level of glycogen was an interesting finding since it has been previously reported that energy deficit attenuates rates of mixed MPS by ~19 % [90]. However, it should be noted that being in an energy deficit state does not necessarily reflects glycogen levels are low. Hence, the total energy available for the cell to undertake its normal homeostatic processes is less.

Summarized, it seems that glycogen availability had no influence on the anabolic effects induced by resistance exercise. However, aforementioned studies on the effects of glycogen availability on resistance exercise-induced anabolic response do not resemble a training volume typically used by resistance-type athletes. Future long-term training studies (~12 weeks) are needed to find out whether performing resistance exercise with low glycogen availability leads to divergent skeletal muscle adaptations compared to performing the exercise bouts with replenished glycogen levels.

### Role of glycogen availability with concurrent training

To date, many sports such as soccer, volleyball, tennis or swimming require a phenotype which features a high oxidative capacity and a sufficient amount of strength/power in order to achieve maximal performance. To develop both endurance capacity and strength/power simultaneously, a targeted training model must include both endurance and resistance exercise, known as concurrent training. Concurrent training does improve both endurance capacity and muscular strength/power, although numerous studies have revealed that strength development is compromised with concurrent training compared to resistance exercise alone [91–94], a phenomenon known as the “interference” effect. Vice versa, the effect of resistance exercise on endurance performance and VO_2max_ appears to be marginal [95, 96]. However, some studies reported compromised gains in aerobic capacity with concurrent training compared to endurance exercise alone [97, 98]. Following the work of Hickson et al. [99], numerous studies have investigated the effect of concurrent training on skeletal muscle adaptations. Although the existing human data on acute molecular events is insufficient to entirely explain the impaired strength/power gains for the concurrent athlete compared with either exercise modality alone, it has been hypothesized that the exercise-induced signaling antagonism may play a role herein. Since a detailed analysis on the interference effect associated with concurrent training is beyond the scope of this review, we refer the reader to expert reviews on the interference effect seen with concurrent training (Baar et al. (2014b); Fyfe, Bishop, & Stepto (2014); Perez-Schindler et al.(2015) [100–102]. Briefly, the most hypothesized mechanism blameworthy for the impaired strength/power gains seen with concurrent training is the impaired upregulation of the P13K-AKT → mechanistic target of rapamycin complex-1 (mTORC1) signaling pathway [89, 102, 103]. It is thought that endurance exercise results in an activation of AMPK, which inhibits the mTORC1 signaling via tuberous sclerosis protein (TSC), and this will eventually suppress MPS resulting in a negative net protein balance. In addition, a higher contractile activity also results in a higher calcium flux, which decreases peptide-chain elongation via activation of eukaryotic elongation factor-2 kinase (eEF2k) leading to a decreased MPS [89, 102, 103]. However, whether the exercise-induced acute interference between AMPK and mTORC1 entirely explains the blunted strength gains seen with concurrent training is to date obscure.

To optimize skeletal muscle adaptations and performance, nutritional strategies for both exercise modes should differ. Indeed, it was recently proposed that, when practicing endurance and resistance exercise on the same day, the endurance session should be performed in the morning in the fasted state, with ample protein ingestion [102]. While the afternoon resistance exercise session should be conducted only after carbohydrate replenishment with adequate post-exercise protein ingestion [102]. Furthermore, whether such a nutritional strategy leads to improved performance compared to general recommendations for carbohydrate and protein intake remains elusive. Interestingly, it has been demonstrated that a resistance exercise session subsequently after low-intensity endurance, non-glycogen depleting session could enhance molecular signaling of mitochondrial biogenesis induced by endurance exercise [104]. Furthermore it is currently unclear whether performing resistance exercise with low-glycogen availability affects the acute anabolic molecular events and whether the effects of these responses possibly result in improved or impaired training adaptation.

Furthermore, whether low-glycogen availability during the endurance bout amplifies the oxidative resistance exercise induced response remains to be investigated. It seems that both modes of exercise in a low glycogen state as part of a periodized training regime are interesting in terms of acute expressions of markers involved in substrate utilization and oxidative capacity. However, on the other hand, a sufficient amount of glycogen is essential in order to meet the energetic demands of both endurance and resistance exercise.

Most existing information on nutrition and concurrent training adaptation is derived from studies where subjects performed exercise in the fasted state [104–108]. Coffey and colleagues investigated the effects of successive bouts of resistance and endurance exercise performed in different order in close proximity on the early skeletal muscle molecular response [76]. Although the second exercise bout was performed with different levels of skeletal muscle glycogen content, the subsequent effects on Akt, mTOR and p70 signaling following the second exercise bout remained the same. Prospective long-term concurrent training studies may help to understand the complexity of the impaired adaptation with concurrent training and further determine to what extend the acute signaling antagonism contributes to this. Moreover, the role of nutritional factors in counteracting the interference effect remains to be further elucidated.

### Research perspectives

In this review we summarized the role of glycogen availability with regard to performance and skeletal muscle adaptations for both endurance and resistance exercise. Most of the studies with low-glycogen availability focused on endurance type training. The results of these studies are promising if the acute molecular response truly indicates skeletal muscle adaptations over a prolonged period of time. Unfortunately, these results on low-glycogen availability may be biased because many other variables including training parameters (time, intensity, frequency, type, rest between bouts) and nutritional factors (type, amount, timing, isocaloric versus non-isocaloric placebo) varied considerably between the studies and it is therefore difficult to make valid inferences. Furthermore, the majority of the studies with low glycogen availability were of short duration [18] and showed no changes [11–17], or showed, in some cases decreases in performance [109]. Nevertheless, reductions in glycogen stores by manipulation of carbohydrate ingestion have shown to enhance the formation of training-induced specific proteins and mitochondrial biogenesis following endurance exercise to a greater extent than in the glycogen replenished state [11–16, 18, 68].

For resistance exercise, glycogen availability seemed to have no significant influence on the anabolic effects induced by resistance exercise when MPS was measured with the stable isotope methodology. However, the exercise protocols used in most studies do not resemble a training volume that is typical for resistance-type athletes. Future long-term training studies (~12 weeks) are needed to investigate whether performing resistance exercise with low glycogen availability leads to divergent skeletal muscle adaptations compared to performing the exercise bouts with replenished glycogen levels.

The role of glycogen availability on skeletal muscle adaptations and performance needs to be further investigated. In particular researchers need to examine glycogen availability when endurance and resistance exercise are conducted concurrently, for example, on the same day or on alternating days during the week. To date, only a few studies have investigated the interactions between nutrient intake and acute response following a concurrent exercise model. We recommend that future research in this field should focus on the following questions:

- What is the impact of performing one of the exercise bouts (endurance or resistance) with low glycogen availability on response of markers of mitochondrial biogenesis of the subsequent (endurance or resistance) exercise bout?
- Does the resistance exercise bout need to be conducted with replenished glycogen stores in order to optimize the adaptive response when performed after a bout of endurance exercise?
- Is nutritional timing within a concurrent exercise model crucial to maximize skeletal muscle adaptations following prolonged concurrent training?

## Conclusions

To conclude, depletion of muscle glycogen is strongly associated with the degree of fatigue development during endurance exercise. This is mainly caused by reduced glycogen availability which is essential for ATP resynthesis during high-intensity endurance exercise. Furthermore, it is hypothesized that other physiological mechanisms involved in excitation-contraction coupling of skeletal muscle may play a role herein.

On the other hand, the low glycogen approach seems promising with regard to the adaptive response following exercise. Therefore, low glycogen training may be useful as part of a well-thought out periodization program. However, further research is needed to further scrutinize the role of low glycogen training in different groups (e.g. highly trained subjects) combined with different exercise protocols (e.g. concurrent modalities), to develop a nutritional strategy that has the potential to improve skeletal muscle adaptations and performance with concurrent training.
